# Retraction: Spinal Cord Stimulation in Complex Regional Pain Syndrome: A Narrative Review

**DOI:** 10.7759/cureus.r256

**Published:** 2026-10-27

**Authors:** John Salib, Mark Salib, Lisa M Tran, David K Sum

**Affiliations:** 1 School of Medicine, St. George's University School of Medicine, St George's, GRD; 2 School of Medicine, St George's University School of Medicine, St George's, GRD; 3 Department of Family Medicine, Humboldt Park Health, Chicago, USA; 4 Department of Anesthesiology, Community First Medical Center, Chicago, USA

This article has been retracted by the Editor-in-Chief. A post-publication review found that a number of statements are attributed to sources that are not in the reference list or that do not support them. The authors acknowledged the errors and agree with the decision to retract.

